# What is desirable care in the opinion of formal and informal caregivers in nursing‐home care for patients with dementia?

**DOI:** 10.1002/nop2.122

**Published:** 2018-01-30

**Authors:** Margreeth van Dijk, Bianca I. Buijck

**Affiliations:** ^1^ Erasmus University Rotterdam Rotterdam the Netherlands; ^2^ Erasmus MC University Medical Center Rotterdam the Netherlands; ^3^ Rotterdam Stroke Service Rotterdam the Netherlands

**Keywords:** dementia, nursing home care

## Abstract

**Aim:**

To examine care characteristics related to desirable care as reported by formal and informal caregivers in Dutch Psycho‐geriatric nursing homes for patients with dementia.

**Design:**

Qualitative exploratory study.

**Methods:**

The sample consisted of four nursing homes. In each home, semi‐structured interviews were conducted with a manager, a quality advisor or head nurse, a daily care supervisor and an informal caregiver. The findings were analysed by labelling and coding the text fragments.

**Results:**

The 16 semi‐structured interviews contained 60 discussion items. The 16 items that were shared by the four interviewee categories were clustered into the following six major themes: good quality of care; poor quality of care; elements of a vision; extra hands; bureaucracy; and formal caregivers.

**Why is this research needed?**

- Since the start of the study presented in this article, complaints were reported about the nursing‐home care for patients with dementia, which resulted in newspaper rankings of the best and the poorest homes. It is important to be aware of those complaints.
- According to these rankings, some nursing homes perform very well, some have a poor performance and the majority show average performance. Pondering this wide range of judgements on the quality of care, the following question arises: what are the characteristics that determine the positive appreciation of formal caregivers, the patients and their informal caregivers?

**What are the key findings?**

- The six care themes that arose in the interviews were: good quality of care; poor quality of care; elements of a vision; extra hands; bureaucracy; and formal caregivers.
- Initial evidence supports the validity of the six characteristics as themes to be discussed in the nursing‐home care for patients with dementia.
- Discussion between formal caregiver, informal caregiver and the patient about the patients' needs is deemed necessary.

**How should the findings be used to influence policy/practice/research/education?**

- Attitudes of caregivers may be subject to change if caregivers are aware of complaints that informal caregivers express.
- The quality of care should be evaluated in structured discussions by the formal caregivers and the informal caregivers of patients with dementia, with reference to individual needs.
- Training and education, according to the theme quality of care, may be necessary to help (informal) caregivers manage individual needs of patients.

## 1 INTRODUCTION

Nursing homes in the Netherlands provide psycho geriatric care to patients with dementia and other disorders in the function of the brain. The care in the nursing homes is subsidized by the national government, with the patient making a financial contribution as well. There are two kinds of nursing homes: small‐scale nursing homes and large‐scale nursing homes. The organization of care in small‐scale nursing homes differs from the care in large‐scale homes. In small‐scale nursing homes, the formal caregiver is responsible for a group of patients and their family representatives. In large‐scale nursing homes a group of formal caregivers is responsible for all the patients and the care is delivered using a task‐centered approach. In 2008, 36,934 patients with dementia were admitted to 368 small‐scale and large‐scale nursing homes in the Netherlands. The mean age of this group was 83 years (Captise_VVT, 2010; De Klerk, 2011; den Draak, 2010; Pot & de Lange, 2010). Besides the care provided by the homes, patients also depend on the care given by their family representatives (informal caregivers). Alzheimer's disease International stated in its 2013 report that in the UK, 80% of older people in nursing homes have dementia (Prince et al., 2013). On 1 January 2015 in the Netherlands, 82,000 patients aged over 65 with dementia were eligible on medical grounds for admission to a nursing home (CIZ, 2015). The Dutch sector association (ActiZ) reported in 2017 that there are 240,000 employees in the sector and 110,000 residents in nursing homes (van Montfort, 2017).

### 1.1 Background

In the Netherlands, the Health Inspectorate (Inspectie Gezondheidszorg, IGZ) collects and provides data on care in nursing homes via their website “Choose better” (http://www.kiesbeter.nl/) and has recently also started doing so via the Patients' Federation (Nederlandse Patiënten Consumenten Federatie, 2015). Nursing‐home patients or their informal caregivers fill in a questionnaire – the consumer quality index (CQ‐I) – with questions about their experience with the care provided in the nursing home of residence (The, Keizer, Paans, & Boekholdt, 2010). The CQ‐I list contains 90 questions on topics such as respect, attention and the patient's privacy. The survey is carried out every second year. A second questionnaire – Medical Indicators, (ZI) – is completed yearly by the formal caregivers in the nursing homes. The ZI instrument rates the opinions of customers (patients and their family) and the organization. The ZI questionnaire is based on 18 aspects relevant to the quality of care, such as the use of antidepressants or sedatives and restraint use. The Dutch Health Inspectorate receives the two sets of measurements (CQ‐I and ZI) where formal and informal caregivers are asked to describe the nursing‐home care provided (Zuidgeest, 2011). Nonetheless, some authors debate that the judgement given by the informal caregivers about quality interventions, can be influenced by dissatisfaction (Winters, 2014). The Inspectorate stopped using the ZI survey in 2014 (BTSG, 2015) and internationally, the interRAI instrument has been used in the past few years. It has now been implemented in the Netherlands as the Resident Assessment Instrument (Hirdes et al., 2008; Holtkamp, Kerkstra, Ooms, van Campen, & Ribbe, 2001). The interRai instrument measures the quality of long‐term care (Carpenter & Hirdes, 2013).

Recent evidence suggests that the working conditions of formal caregivers are an important aspect in delivering qualitatively good care (Gibson, Carter, Helmes, & Edberg, 2010; Holtkamp, Kerkstra, Ribbe, van Campen, & Ooms, 2000; Inspectorate for the Health Care, 2003; Lubart et al., 2004). Furthermore, similar signals were found in the US, with nurses experiencing poor working conditions due to aggressive behaviour (from verbal threats to physical assault) from residents on an almost daily basis (Lindbloom, Brandt, Hough, & Meadows, 2007). High quality care also depends on the quality of an organization (Hardjono & Bakker, 2011; van het Erve, Gorter, Berge, & Bakker, 2004). It is associated with the aim to provide safety, support and warmth with compensation for what is missed (Pot & de Lange, 2010). It is also associated with the transparency and vision of the organization regarding patients with dementia and the focus of the care on the provision of customer choice information (ActiZ, 2012). Signals of poor quality care include errors in the form of abuse (Bakker, 2002), personnel shortages, staff being obsessed with the technical nursing task, or the nurses considering the informal caregivers of patients with dementia as a source of additional work pressure (Thys, 2003 – 2004). Regarding preventive policies, it is necessary to focus on supervision to identify violations of elderly people's rights and psychological and physical abuse, as well as on building organizational cultures that respect ethical principles (Buzgova & Ivanova, 2009).

The data from the two questionnaires (opinions and merit rating) were analysed by a Dutch newspaper (*De Volkskrant*, Trommelen & de Visser, 2010), which resulted in a ranking of nursing homes. According to this ranking, some nursing homes perform very well, some have a poor performance and the majority show average performance (Lindbloom et al., 2007; Peeters, van Beek, Meerveld, Spreeuwenberg, & Francke, 2010). Pondering this wide range of judgements on the quality of care and the limited information that was found about the participation of the informal caregivers, the following question was asked: what are the characteristics that determine the positive appreciation of formal caregivers, the patients and their informal caregivers? And how can these characteristics be facilitated by the nursing homes?

Therefore, the aim of this study is:

- to examine care characteristics related to desirable care as reported by formal and informal caregivers in Dutch Psycho‐geriatric nursing homes for patients with dementia;
- to explore the characteristics that determine the positive appreciation of formal caregivers, the patients and their informal caregivers.

## 2 THE STUDY

Four databases were searched – the Erasmus University library, Cochrane, the Dutch Royal library (Koninklijke Bibliotheek) and PubMed – for instruments used nationally and internationally for measuring the quality of care. Literature on family participation in nursing‐home care was also analysed. The reason for the literature study was to discover which instruments were up to date. The terms for the literature searches were: Psychogeriatric And dement* AND care AND nursing home AND elderly AND measuring AND instrument 1997–2011. Table A1 summarizes the 17 articles that met the inclusion criteria (the search terms). In this context, the newspaper's list of 350 publicly subsidized nursing homes was used for a selection of a sample.

### 2.1 Design

The design of this qualitative, exploratory study was influenced by the results of the ranking of 350 nursing‐homes reported by the newspaper, which used the CQ‐I and ZI questionnaires (Trommelen & de Visser, 2010). The newspaper mentioned the dissatisfaction of residents and their families with the nursing‐home care for patients with dementia in their conclusion. Two nursing homes that were classified as among the best and two that were classified as among the poorest were contacted. Semi‐structured interviews with the formal and informal caregivers of those nursing‐ homes were used to explore their opinions about the nursing care. An interview guide was developed, with questions and topics, but the semi structured nature of the interview allowed interviewer and interviewee to bring up new ideas during the interview.

### 2.2 Data collection

The above‐mentioned 17 articles discussing measuring instruments were unable to provide an individual conclusion or understanding at the family level of a patient with dementia. At the time the research was carried out, the Dutch instrument Prestatie‐overzicht Kwaliteitskader VV&T was the most up‐to‐date assessment method for nursing homes and included the CQ‐I (VV&T Stuurgroep Kwaliteitskader, 2011); it therefore became the starting point for the study with the semi‐structured interviews.

The aim of the semi‐structured interviews was to elicit the participants' understanding of dementia care. Three categories of formal caregivers and one informal caregiver involved in the care for patients with dementia were interviewed: (i) a manager [M]; (ii) a quality advisor or head nurse [Q]; (iii) a daily care supervisor [S]; and (iv) an informal caregiver [I] (usually a relative, as well a member of the patients' board and chosen by the manager). Thus 16 caregivers were interviewed in total. These interviews with the caregivers took about 60 min each and questions were similar for all categories. The semi‐structured interviews started with an open question: “In your opinion, what is the dependent or independent care variable that would let your nursing home stay in this position in the classification by the newspaper?”

### 2.3 The interview

In this study, two nursing homes that were classified as among the best and two that were classified as among the poorest were selected for an interview visit by the researcher. The management of the four homes were approached and asked to participate in the interviews. All participants were asked for consent. The choice for interviews was made as a way of having objective face‐to‐face contact with the formal and informal caregivers of the patients with dementia (Glaser & Strauss, 1967). In December 2011, semi‐structured interviews were held to obtain “free‐format” information about the desirable care.

### 2.4 Data analysis

A transcription of all interviews was documented and sent to the interviewees concerned for validation. After their confirmation, the findings were processed by labelling and coding the text fragments. In each sentence of the transcriptions, care aspects were identified and labelled. The informal caregivers were considered the best source regarding desirable care, therefore the method of labelling started with their interview transcription. This was followed by the formal caregivers' transcriptions. A characteristic was considered meaningful if there was consensus among all categories. All analyses were carried out and the search function in Word and Excel was used to find and bundle the characteristics. The 16 semi‐structured and confirmed interview transcriptions provided 60 discussion items. Every sentence was assigned a characteristic. In the end, 60 characteristics were identified. Of these 60 characteristics, 16 appeared in the transcriptions of all four categories of interviewed persons. The analysis continued with these 16 characteristics where there was consensus among all categories of caregiver.

### 2.5 Ethical consideration

After approval by the management and the “patients' council” of each nursing home, all formal and informal caregivers were informed about the purpose of the study and the interview. After this information was received, the participants gave their informed consent.

## 3 RESULTS

The frequency with which the characteristics were mentioned in the interviews is shown in Table A2, in the appendix. The labelled characteristics are listed in the first column. Subsequent columns indicate the number of times the characteristic was mentioned in the transcription by the interviewed persons. The managers of the best homes are indicated by M1 + M3 and the managers of the poorest homes by M2 + M4, the quality advisors or head nurses by Q1 + Q3 and Q2 + Q4, the daily care supervisors by S1 + S3 and S2 + S4 and the informal caregivers of the patients with dementia by I1 + I3 and I2 + I4. The 16 shared characteristics were clustered into six major themes (a–f, see Table A3, appendix) as a way of understanding the situation in the nursing homes.

Table A4 in the appendix shows the combined characteristics of a similar nature from the interview transcription, with the differences between the best and poorest nursing homes. In each row the four homes are listed, each with their four interviewee categories. In the first column the 16 characteristics originally identified are grouped into the six summarizing themes. Subsequent columns indicate the number of times the theme was mentioned in the transcriptions of the interviewed persons, combining the two best homes and the two poorest homes.

The characteristics of the best and the poorest nursing homes were examined for differences and similarities. Characteristics of a similar nature from the best and the poorest homes were noted, as well were the opinions of the interviewed persons. The greatest differences appear in the frequency of references by quality advisors or head nurses according to poor quality of care and bureaucracy. The poorest homes reported poor quality 38 times, and the best homes 3 times. Bureaucracy was reported 8 times (best nursing homes) compared to 35 times (poorest homes).

To get a better understanding of what is seen as important in the best nursing homes, a selection of remarks of the six major themes (a–f) in the interview transcriptions is given below. We also added the citations concerning the original 16 characteristics and – between brackets – the interviewee category concerned: [M] a manager; [Q] a quality advisor or head nurse, [S] a daily care supervisor and [I] an informal caregiver of a patient with dementia. The choice of the characteristic and category citations was made by the researcher, and was based on the importance of the characteristic and differences between caregiver categories. In this way the variation in citations and between categories was shown:

- Good quality of care
  Attention: [I] “The nurses have to work very fast. They are busier than before with all kinds of things at the expense of the attention for the patients.” Attitude: [Q] “According to research done in the organization, important factors are: keep responsibility in the teams, let team members alert one another to ensure the right attitude.” Satisfaction: [M] “The patients want to live in a nice and safe environment, to eat well and have clean rooms. The families give these elements a high score.”
- Poor quality of care
  Aggressive patients: [S] “As the supervisor responsible for the daily care, we can give less attention to the patients. As an example: when a patient is aggressive they cannot walk outside. We have to give them medication.” Inspection: [I] “It is not known what kind of performance indicators the Inspectorate of the Ministry of Health used in this nursing home. Based on my professional experience, the Inspectorate monitors files. They check if the right papers are in the files with regard to events and decrees of the court.” Complaints: [M] “Patients do have a culture of complaining, but they do not call the complaints desk.”
- Elements of a vision
  Small‐scale: [Q] “Important reasons for establishing small‐scale nursing homes are, for example, a feeling of engagement among the family and less staff absenteeism.” Multi‐Disciplinary Consultation: [S] “The family cannot attend the Multi‐Disciplinary Meeting of professionals to give their input. The daily care supervisor talks to the informal caregiver of the patient with dementia before and after the Multi‐Disciplinary Meeting.” Admission: [I] “Mother would have liked to live independently, but she was diabetic and wasn't eating. It took a long time before she signed for the admission.” Vision: [Q] “To give structure to the patients for their safety, we want the patients to be themselves.” Care plan: [S] “In the first contact between the doctor and the family, we started with discussing the care plan.”
- Extra hands
  Family: [M] “In our institution, we have critical care family assistance; for example a daughter moved to this region and admitted her mother to this institution nearby. She helps a lot.” Volunteers: [I] “Not everybody can cope with the fact that the same thing is said three times in 5 min.”
- Bureaucracy
  Data: [M] “The way the Ministry looks at the data of a care home differs from the way patients experience their well‐being. The Inspectorate is more likely to plan their audits when there has been an incident in a facility in another part of the Netherlands.” Classification:[Q] “Because of the successful classification, the project “small scale living” was ended and the budget was cut, which is a pity for the facility. They cannot give the same attention to the patients as they did before in the project.”
- Formal caregivers
  Education: [Q] “Within the organization, an education program has been developed, which has not yet been implemented in the unit included in the ranking.”

Citations about good quality of care are given in Table A5, appendix. The table gives the citations of the interview transcriptions in the four interviewee categories concerning good quality care in the best and the poorest homes.

When comparing the remarks by the interviewees in the best homes and those in the poorest homes, we found some differences. The number of times family is mentioned by the health‐care workers responsible for the daily care in the two best homes is 14 and 11 times, respectively, compared with 22 and twice, respectively, in the poorest homes. The managers in both the best and the poorest homes also often mentioned the importance of family, 13 and 3 times, respectively, in the best homes compared with 11 and 6 times, respectively, in the poorest homes.

## 4 DISCUSSION

This study aimed to find characteristics for desirable care as reported by formal and informal caregivers. Six themes concerning desirable care emerged based on 16 characteristics used in the interview transcriptions. The six themes are: good quality of care; poor quality of care; elements of a vision; extra hands; bureaucracy; formal caregivers. These themes emerged from the data, however, the judgement of caregivers can be influenced by dissatisfaction (Winters, Kool, Klazinga, & Huijsman, 2014) and this may have influenced characteristics and themes. Nevertheless, these themes may give direction to discussion with informal caregivers about desirable care.

Characteristics of the best and the poorest nursing‐homes were examined for differences and similarities. When comparing the remarks of the interviewees in the best homes and those in the poorest homes, we found some differences. In other studies there were also differences identified, and the judgements of the patient' federation differs to our study‐results in terms of themes. Their assessments of the top five elements of care in the nursing homes concerned the positive characteristics of love, small‐scale, involvement, gardens and more space (Nederlandse Patiënten Consumenten Federatie, 2015). The difference in desirable care between the best and poorest nursing homes may be based on the attention given to the family. This could have several reasons, such as the level of education of the formal caregivers and the lack of time. Research shows that informal caregivers have specific needs that are not always being met at the desirable moment by the formal caregivers, possibly resulting in dissatisfaction. The difference in needs varies according to the types of main informal caregivers (e.g., spouses versus sons/daughters and children‐in‐law) and the living situation of the patients with dementia. It is assumable that informal caregivers need additional information and advice tailored to their specific needs (Afram et al., 2014 ; Peeters et al., 2010).

All interviewees give their personal views about the care given and received. When prior studies on the topic were examined, general information was found but no information about individual stories. It is of great importance that health‐care professionals are open to and listen to the life storytelling of patients with dementia and their informal caregivers (Heggestad & Slettebo, 2015), as it has an important positive influence on their dignity. An individual approach in the care can give more satisfaction to the patients and their informal caregivers.

The two highest ranked organizations participating in this study were proud of their best classification. One of the top‐ranked organizations interviewed mentioned: “The unit is small with 14 beds/patients and received an AGB [General Data Administration – *Algemeen Gegevens Beheer*] code in 2009, which resulted in receiving €90,000 extra above the usual budget”. It was said in the interview that the unit was counted as a separate organization while nowadays “you receive such a code when you have a unit with 25 patients”. The extra money may have given an impulse to quality improvement. The poorest classified home said: “In 2010, the CQ‐I was measured by an independent bureau, but they have forgotten to deliver the outcome”. It seemed that with enough finance and reliable independent organizations the classification and outcome can be improved.

In this study, the national ranking by the newspaper (*Volkskrant*) concerning quality of care in the nursing homes was used (Trommelen & de Visser, 2010). During the study, a year after the first ranking, a second ranking was published in a magazine (Heida, Boonen, & Sonneveld, 2011). Nowadays the nursing‐home evaluation is given as a mean figure (Nederlandse Patiënten Consumenten Federatie, 2015). The organizations can influence the results of the ZI questionnaire by implementing activities, but are dependent on others such as informal caregivers for the CQ‐I outcomes.

A small‐scale location turned out to be the best in the ranking (Trommelen & de Visser, 2011). The organization of care in small‐scale nursing homes is one of the top five criteria of the patient federation (Nederlandse Patiënten Consumenten Federatie, 2015). However, research shows that small‐scale/homelike care environments are not necessarily better care environments than regular nursing homes (Verbeek, Zwakhalen, van Rossum, Kempen, & Hamers, 2013). It seems that quality of life and behaviour of patients, job satisfaction and the motivation of nursing staff are more important than the location (Willemse, Wessel, & Pot, 2015). Further research is needed to collect more individual stories by using the six themes (a–f). The judgement about the care quality should be given by the patient or informal caregiver on an individual level and find its place in the care plan formed by an individual story (Pronk, 2016).

## 5 STRENGTHS AND LIMITATIONS

A strength of the study is that all the interviews were carried out by the same interviewer. Moreover, the results were discussed in peer groups (Winters, Strating, Klazinga, Kool, & Huijsman, 2010) and the data were analysed using both quantitative and qualitative methods.

The research was a continuous exploratory process and has some limitations. An entrance into the nursing‐home organizations in general was difficult at the start. ActiZ was asked to participate as a databank entrance, but is very reticent in sharing information and therefore difficult to approach for an (external) researcher. Also, the national – assessment centre, where they assess if a person is eligible for long‐term care support and which has a lot of data, was not willing to share information. The ranking by the newspaper with the best and poorest nursing homes based on the results of the CQ‐I and ZI became therefore the entrance into the homes (The et al., 2010). A limitation is possible information bias in the responses because the participants in the study (the individuals who represented the four categories) were chosen by the management of the selected high‐ranking or low‐ranking organizations.

## 6 CONCLUSIONS

There are some differences in the overall opinions as reported by formal and informal caregivers on desirable care between two of the best nursing homes and two of the poorest nursing homes. Informal caregivers said that patients want well‐being and the right attitude from formal caregivers, defined as living in pleasant and safe conditions, with healthy food and a clean ambiance. This is a responsibility for all (informal) caregivers involved in the care for patients with dementia. Most patients with dementia are not able to stand up for themselves and are dependent on others to take care of them. Validation of the six themes is necessary to provide insight into formal and informal caregivers' opinions about the desirable care in nursing homes for patients with dementia.

## CONFLICT OF INTEREST

The authors declare that they have no competing interests.

## APPENDIX 1

### 1.1

**Table A1:** The 17 articles that met the inclusion criteria (the search terms)

References	Measuring instrument
Cooney and Mortimer (1995)	Postal questionnaire for the carers of dementia
Arends (1998)	The BOPZ
Dijkstra, Buist, Dassen, and van den Heuvel (1999)	The Care Dependency Scale, CDS
Holtkamp et al. (2001)	The resident assessment instrument
Ettema et al. (2005)	Quality of life instruments used in dementia
Zwakhalen, Hamers, and Berger (2007)	A behavioural pain scale
Achterberg, Pot, Scherder, and Ribbe (2007)	The nottingham health profile
Gerritsen et al. (2008)	The MDS challenging behaviour profile
McCool, Jogerst, Daly, and Xu (2009)	Multidisciplinary reports
Cooper, Maxmin, Selwood, Blanchard, and Livingston (2009)	The Modified Conflict Tactics Scale
Berkhout, Boumans, Mur, and Nijhuis (2009)	“7‐S” model
Acierno et al. (2010)	The National Elder Mistreatment Study
Triemstra, Winters, Kool, and Wiegers (2010)	The consumer quality index long‐term care
Inspectie voor de Gezondheidszorg, (2010)	Vrijheidsbeperking
Heidstra‐Wolke (2011)	Zelf‐check voor verpleeghuisartsen
Pillemer et al. (2011)	Research‐to‐practice consensus
VV&T Stuurgroep Kwaliteitskader (2011)	Kwaliteitskader VV&T

**Table A2:** The frequency with which the characteristics were mentioned in the interviews

	By institution and discipline→	M1	Q1	S1	I1	M2	Q2	S2	I2	M3	Q3	S3	I3	M4	Q4	S4	I4
	Characteristics ↓																
1	Attention		1	2	3	1		2	3	1	2	2	2			1	2
2	Aggressive patient			1	1	1	3	1			1						
3	Attitude		3				1	2	1	1	1	1	1	2	4		1
4	Data	1	4		1	2	16	'1		2	1	1	1	1	14		
5	Inspection	7		1	6	5	7	5	2	3		3	1	2	9	1	2
6	Complaint	3	1	2	1	1	7	1	1	1	1	4	1	4	12	2	3
7	Small‐scale		3		1	2				9	4	2		2	1	2	
8	Family	13	5	14	2	11	1	22	7	3	3	11	3	6	5	2	1
9	Multi‐disciplinary consultation		1	4		2		2	1	2		2			3	2	
10	Admission	5	1	2		2		4				7	7	1		1	
11	Classification	3	1	2		1	1	1	3	1	2	2		5	4		2
12	Education	3	3	4		4	2	2	4	5	1	2		2	2	3	
13	Satisfaction	2	6	1	1		1		1	1	1		1		2		
14	Vision	1				1	2	1		1	3	2			1		
15	Volunteers	3			2	5	1	8			2					2	
16	Care plan	2	6	8		4	2	7			3	1	2		4		1

**Table A3:** The shared characteristics clustered into major themes

16 characteristics	►	Six major themes
1) Attention, 2) Attitude, 3) Satisfaction		A) Good quality of care
4) Aggressive residents, 5) Inspection, 6) Complaint		B) Poor quality of care
7) Small‐scale, 8) Multi‐Disciplinary Consultation, 9) Admission, 10) Vision, 11) Care plan		C) Elements of a vision
12) Family, 13) Volunteers		D) Extra hands
14) Data, 15) Classification		E) Bureaucracy
16) Education		F) Formal caregivers

**Table A4:** The combined characteristics of a similar nature showing the difference

By institution and discipline →	M1 + M3	M2 + M4	Difference	Q1 + Q3	Q2 + Q4	Difference	S1 + S3	S2 + S4	Difference	I1 + I3	I2 + I4	Difference
Characteristics ↓												
Attention	1	1	0	3		Minus 3	4	3	Minus 1	5	5	0
Attitude	1	2	Plus 1	4	5	Plus 1	1	2	Plus 1	1	2	Plus 1
Satisfaction	3		Minus 3	7	3	Minus 4	1		Minus 1	2	1	Minus 1
A. Good quality of care	5	3	Minus 2	14	8	Minus 6	6	5	Minus 1	8	8	0
Aggressive patient		1	Plus 1	1	3	Plus 2	1	1	0	1		Minus 1
Inspection	10	7	Minus 3		16	Plus 16	4	6	Plus 2	7	4	Minus 3
Complaint	4	5	Plus 1	2	19	Plus 17	6	3	Minus 3	2	4	Plus 2
B. Poor quality of care	14	13	Minus 1	3	38	Plus 35	11	10	Minus1	10	8	Minus 2
Small‐scale	9	4	Minus 5	7	1	Minus 6	2	2	0	1		Minus 1
Multi‐disciplinary consultation (MDO)	2	2	0	1	3	Plus 2	6	4	Minus 2		1	Plus 1
Admission	5	3	Minus 2	1		Minus 1	9	5	Minus 4	7		Minus 7
Vision	2	1	Minus 1	3	3	0	2	1	Minus 1			0
Care plan	2	4	Plus 2	9	6	Minus 3	9	7	Minus 2	2	1	Minus 1
C. Vision	20	14	Minus 6	21	13	Minus 8	28	19	Minus 9	10	2	Minus 8
Family	16	17	Plus 1	8	6	Minus 2	25	24	Minus 1	5	8	Plus 3
Volunteer	3	5	Plus 2	2	1	Minus 1		10	Plus 10	2		Minus 2
D. Extra hands	19	22	Plus 3	10	7	Minus 3	25	34	Plus 9	7	8	Plus 1
Data	3	3	0	5	30	Plus 25	1	1	0	2		Minus 2
Classification	4	6	Plus 2	3	5	Plus 2	4	1	Minus 3		5	Plus 5
E. Bureaucracy	7	9	Plus 2	8	35	Plus 27	5	2	Minus 3	2	5	Plus 3
Education	8	6	Minus 2	4	4	0	6	5	Minus 1		4	Plus 4
F. Formal caregivers	8	6	Minus 2	4	4	0	6	5	Minus 1		4	Plus 4

**Table A5:** Citations about good quality of care

	Best homes		Poor homes
M1	Excellent care that includes personal communication; Constructively critical families, they help a lot; Families live in the area; Few changes in personnel; Trainees bring “breath of fresh air”; Short lines of communication and if there are problems, the family is called with mutual clear expectations.	M2	Every day the personnel of the organization are busy with quality. You need to feel quality in your bones; it is an attitude and about being open to change. Formal caregivers give good care, are enthusiastic and are occasionally allowed to be “naughty” in a responsible way.
Q3	Choose what is best for the patients. Let the patients stay in control and have their own opinion. As a team look at what is possible and find solutions with a willingness to review decisions that have been taken. If you have a good plan to improve care, then you give attention.	Q4	The organization is very transparent and open. Patients' satisfaction is very important in the organization. The risk assessment is documented and discussed with the family in the Multi‐Disciplinary Consultation.
S1	To take care of the patients and care for them in the final stage of life, with whatever food they want. To pay proper attention to the needs of the patients. To get the right balance for the medication and prevention of falls, in consultation with the family. When the doctor visits, the family is called and the necessary information can be discussed. The trainees are taking patients to “go for a walk outside”.	S2	Looking after the wishes and needs of the patients. Complex care of the patients is a task for the qualified personnel (permanent staff). The personnel of the organization are more occupied with the care plan and are monitoring the implementation.
I3	The most important thing is, that my mother has good personal hygiene, that the food is good, that she is in friendly surroundings where they give her care, put an effort into activities and have the right attitude. Mother says “They are very good to me.”	I4	Most important in the care of my mother‐in‐law are: clean clothes and room, daily showers and individual attention by the caregivers. They promote the positive atmosphere in the group, both for the patients and for the caregivers.
